# Alginate/Fish Gelatin-Encapsulated *Lactobacillus acidophilus*: A Study on Viability and Technological Quality of Bread during Baking and Storage

**DOI:** 10.3390/foods10092215

**Published:** 2021-09-18

**Authors:** Milad Hadidi, Nava Majidiyan, Aniseh Zarei Jelyani, Andrés Moreno, Zahra Hadian, Amin Mousavi Khanegah

**Affiliations:** 1 Department of Organic Chemistry, Faculty of Chemical Sciences and Technologies, University of Castilla-La Mancha, 13071 Ciudad Real, Spain; andres.moreno@uclm.es; 2 Department of Veterinary Medicine, Urmia Branch, Islamic Azad University, Urmia 57169-63896, Iran; Navamajidian94@gmail.com; 3 Food Control Laboratory, Department of Food and Drug, Shiraz University of Medical Science, Shiraz 71348-14336, Iran; Aniseh.Z.jelyan@gmail.com; 4 Department of Food Technology Research, Faculty of Nutrition Sciences and Food Technology, National Nutrition and Food Technology Research Institute, Shahid Beheshti University of Medical Sciences, Tehran 19395-4741, Iran; z_hadian@sbmu.ac.ir; 5 Department of Food Science and Nutrition, Faculty of Food Engineering, University of Campinas, São Paulo 13083-852, Brazil

**Keywords:** bread, fish gelatin, probiotic, encapsulation, viability

## Abstract

In the present study, *Lactobacillus acidophilus* LA-5 was microencapsulated in sodium alginate, followed by fish gelatin coating (0.5, 1.5, and 3%). The survival of *L. acidophilus* in bread before and after encapsulation in alginate/fish gelatin during the baking and 7-day storage was investigated. Moreover, the effect of alginate/fish gelatin-encapsulated *L. acidophilus* on the technological properties of bread (hardness, staling rate, water content, oven spring, specific volume, and internal texture structure) was evaluated. Compared with control (free bacteria), encapsulated *L. acidophilus* in alginate/fish gelatin showed an increase in the viability of bread until 2.49 and 3.07 log CFU/g during baking and storage, respectively. Good viability of (10^6^ CFU/g) for probiotic in encapsulated *L. acidophilus* in alginate/fish gelatin (1.5 and 3%, respectively) after 4-day storage was achieved. Fish gelatin as a second-layer carrier of the bacteria had a positive effect on improving the technical quality of bread. Furthermore, the staling rate of bread containing encapsulated *L. acidophilus* alginate/fish gelatin 0.5, 1.5, and 3% decreased by 19.5, 25.8, and 31.7%, respectively. Overall, the findings suggested encapsulation of *L. acidophilus* in alginate/fish gelatin capsule had great potential to improve probiotic bacteria’s survival during baking and storage and to serve as an effective bread enhancer.

## 1. Introduction

Bread is an innovative field in the probiotic food sector and has attracted rising interest in research. Due to the high temperatures during the bread baking process, probiotics in bread are challenging [1]. Probiotics are live bacteria and yeasts promoted as having health benefits to the host when consumed adequately. Moreover, these microbes must survive the processing and storage of food, as the presence of large numbers of unstressed and viable microbial cells at the time of using them is responsible for the health benefits. The probiotic counts between 10^6^ and 10^9^ CFU/g are universally admitted in the food [2,3].

A promising strategy to enhance probiotic bacteria’s survival is to encapsulate probiotic cells in a protective capsule. The viability of probiotics in a solid matrix during heat processes is influenced by the composition of the matrix [4]. Zhang et al. [5] encapsulated *Lactobacillus plantarum* in four encapsulating materials, including reconstituted skim milk, Arabic gum, maltodextrin, and inulin. They found that the survival of probiotics in bread during baking was significantly influenced by the approach used to incorporate strains and physicochemical properties of coating materials. In a similar study, coated alginate-based microcapsules with an egg stearic acid/albumen matrix were used to encapsulate *Lactobacillus acidophilus*. The viability of encapsulated bacteria was found higher during the baking of bread [6]. Encapsulation of lactobacilli in alginate beads has been found to enhance their thermal stability and increase survival during heating and storage [7,8]. In this regard, present studies mainly focused on the encapsulation of probiotics and their viability on bread. In contrast, investigations on the impact of encapsulated probiotics in biopolymers such as alginate and fish gelatin on staling and technological quality of bread during storage remain scarce.

Hydrocolloids are an excellent ingredient for improving the technical quality of bread. Among them, one of the best materials for making hydrogels is fish gelatin. Compared to mammalian gelatins, fish gelatin does not induce bovine spongiform encephalopathy and has no religious limitations. Moreover, fish gelatin is microbiologically stable and derived from byproducts and waste from the fisheries and aquaculture industry, thus decreasing environmental pollution and production costs [9]. In addition, fish gelatin improves the cohesivity and stability of the network structure through electrostatic interactions and stable intermolecular hydrogen bonds between the cationic amine group in fish gelatin (-NH3^+^) and the carboxylate group in alginate (-COO^-^) [10].

As a complex process, bread staling is a critical quality factor for wheat bread, including moisture loss and textural changes. The bread staling process involves several mechanisms such as migration of moisture, starch–gluten interaction, and retrogradation of starch and amylopectin [11]. Water migration and hardness can also significantly limit bread quality during storage and shelf life of baked products [12].

This investigation aims to create new functional food to improve the viability of *L. acidophilus* LA-5 during bread baking and storage using microencapsulation in sodium alginate, followed by fish gelatin-coating (0.5, 1.5, and 3%). Moreover, adding alginate/fish gelatin-encapsulated *L. acidophilus* on the technological qualities of bread such as hardness, staling rate, water content, oven spring, specific volume, and internal texture structure during 7 days of storage was studied.

## 2. Materials and Methods

### 2.1. Preparation of Probiotics Bacterial Cell

The *Lactobacillus acidophilus* (LA-5) probiotic strain was provided by Christian Hansen (Horsholm, Denmark). MRS broth (Neogen Corporation, Lansing, MI, USA) was employed to prepare the probiotic culture as the growth medium. A single microorganism colony was inoculated in the sterile growth medium (10 mL) and pre-cultured at 37 °C for 12 h. Afterward, 1% *v*/*v* inoculum of *L. acidophilus* were sub-cultured in MRS broth (100 mL) at 37 °C without agitation for 24 h. The *L. acidophilus* cell was harvested and washed with sterile 0.9% saline solution by centrifugation at 4 °C and 10,000× *g* for 10 min (BXT-H165A, Baxit, China).

### 2.2. Encapsulation in Alginate/Fish Gelatin Matrix

*L. acidophilus* was microencapsulated in sodium alginate according to the emulsion method defined by Moghanjougi et al. [13] with minor modifications. Firstly, sodium alginate (2% *w*/*v*) and fish gelatin (0.5, 1.5, and 3% *w*/*v*) solutions were separately prepared in deionized water and sterilized at 121 °C for 15 min (15 psi). Then, 10 mL of microbial suspension was mixed with 40 mL of sodium alginate solution in a sterile condition, then added steadily by sterile syringe into a sterilized solution containing 198 g of rapeseed oil and 2 g of Tween 80 and mixed using a magnetic stirrer (3500, Anzeser, Tokyo, Japan) at 750 rpm for 20 min. Next, sterilized calcium chloride solution (80 mL, 0.1 M) was added to the mixture by syringe as a dropper, and then the solution was blended on a magnetic stirrer for 10 min at 100 rpm. Sterile peptone water (80 mL) was added for separating the phases, and the emulsion was stabled for 30 min. After the sedimentation of formed beads, the microcapsules were divided by centrifugation at 400× *g* for 10 min at 4 °C, and the oily fraction was eliminated. The obtained beads were washed twice with sterile physiology serum (0.9%) to remove residuals. Afterward, microcapsules were immersed in 10 mL of fish gelatin solution (0.5, 1.5, and 3% *w*/*v*) and blended for 30 min at 500 rpm, transferred into a sterile glass container, and dried in a lyophilizer (RL-50DG, Really, Henan, China) at −56 °C for 24 h. Finally, prepared capsules were kept in a refrigerator.

### 2.3. Breadmaking

Bread samples were produced according to the following formulation: 200 g of wheat flour, 8 g of sugar, 3 g of salt, 2 g of instant yeast, 6 g of butter, and UHT skim milk (130 g) with probiotics added. Bread with non-encapsulated bacteria addition was prepared as the control. For dough preparation, the dry ingredients were mixed in a stand mixer (Malka-20, Amasadora, Alicante, Spain) at 40 rpm for 1 min, then milk and probiotic were added and kneaded at 80 rpm for 7 min. After 5 min of resting, the dough was divided into balls of 50 g and proofed at 85% RH and 40 °C in a climate chamber (NT-125, Always here, China) for 60 min. Samples were baked at 175 °C for 6 min in an electric oven (Maxima Deluxe 5, Granada, Spain). Finally, bread was packed in polyethylene bags and kept at room temperature and 55% RH for 7 days in the climate chamber.

### 2.4. Viable Cell Counts of L. acidophilus

Five gram of samples (dough or bread) was aseptically homogenized with sterile peptone water (45 mL, 0.1% *w*/*w*) in a stomacher (400, Seward, AK, USA). Serial dilutions of the suspensions (100 μL) were prepared in sterile peptone water (900 μL), and 100 μL solution was plated onto the MRS agar broth (Neogen, Corporation, Lansing, MI, USA) incorporated with 200 mg/L natamycin (Merck Chemical Co., Darmstadt, Germany). Natamycin was used for inhibiting the yeast’s growth on the MRS agar plate, which did not affect the probiotics’ growth [14]. The plates were counted after incubation at 37 °C for 48 h. The results were expressed as log CFU/g. 

### 2.5. Encapsulation Efficiency, Particle Size, and Zeta Potential of Capsules

The particle size and electrical charge of the capsules were measured by a laser diffraction Mastersizer (Malvern Instruments, Worcestershire, UK) and a Zeta seizer (Malvern Ltd., Malvern, UK), respectively [15].

The encapsulation efficiency was estimated as the percentage of loaded cells in the capsule relative to the initial *L. acidophilus* cells according to the following equation:

Encapsulation efficiency (%) = N/N_0_ × 100

where N represents the viable count of encapsulated cells after releasement and N_0_ is a total number of viable free cells added initially to matrix material during the encapsulation process [16].

### 2.6. Determination of Hardness and Staling Rate

A Brookfield CT3 texture analyzer was employed to determine crumb hardness. A bread slice (thickness of 2.5 cm) was compressed up to 40% deformation with a 38.1 mm acrylic probe at 120 mm/min speed and 1 min of hold time. The hardness is related to the peak force of the first compression cycle. The staling rate of bread samples was estimated as increases in crumb hardness after the final day of storage [11] as follows:

Staling rate = [hardness (day 7 − day 0)/days of storage]

### 2.7. Determination of Moisture Content

The moisture content of samples was measured according to the Approved Method of AACC 44-15.02 [17]. The samples were dried in an oven at 105 °C for 8 h. Moisture content was estimated for fresh bread and after storage (1, 4, and 7 days) as the following formula:

Moisture content (%) = [initial weight − weight after drying/initial weight] × 100

### 2.8. Oven Spring and Specific Volume Index

The difference in dough height before and after baking was considered the oven spring of bread [18]. The baked sample was weighed after 30 min cooling (g), and its volume (cm^3^) was measured according to the rapeseed displacement method. The specific volume (cm^3^/g) was estimated as bread volume/bread weight [19].

### 2.9. Internal Texture Structure Analysis

The image scanner studied the bread’s internal texture structure (Sony 70-DM, Tokyo, Japan) and analyzed it using ImageJ analysis software (ver. 2.3). The cropped images (4 × 4 cm^2^) were converted to 8-bit grayscale binary images with a resolution of 600 dpi to quantify porosity (ratio pore area/16 cm^2^), cell density (cells/cm^2^), and cell average area (ratio pore area/number of cells) [11].

### 2.10. Statistical Analysis

All experimental data were expressed as means ± standard deviations (*n* = 3) after passing the analysis of variance (ANOVA) and Duncan’s multiple range tests using SPSS statistics software (ver. 22.0). *p* < 0.05 was accepted to be statistically significant.

## 3. Results and Discussion

### 3.1. Encapsulation Efficiency, Particle Size, and Zeta Potential of Capsules

The encapsulation efficiency of *L. acidophilus* was 92.3% for the alginate as a single carrier, similar to the results observed by Moghanjougi et al. [13] by 95.5% for *L. acidophilus* and Jouki et al. [16] by 93.1% for *Lactobacillus casei* in alginate beads. The encapsulation efficiency of *L. acidophilus* significantly increased along with the increase of concentration of fish gelatin from 92.3 to 98.68% (Table 1). Some factors like the hydrogen bonding between carboxylate groups of fish gelatin and sodium alginate and good interaction between these biopolymers could increase encapsulation efficiency [20,21]. The range of particle size of encapsulated *L. acidophilus* in alginate/fish gelatin was from 289.8 to 736.5 μm with a relatively narrow range of size distribution. The particle size of encapsulated *L. acidophilus* in alginate/fish gelatin ranged. The size of capsules is an important physical property that affects the bioactivity properties and its stability [22]. The results indicate that the size of the *L. acidophilus* capsules increased after coating by fish gelatin. Compared to smaller microcapsules, larger capsules provide higher protection against the core material in the food systems [16]. The zeta potential values of encapsulated *L. acidophilus* in alginate/fish gelatin ranged from −13.5 to −26.1 mV (Table 1). The high zeta potential of colloidal particles improves the physical stability and the electrostatic repulsion force of the system. Several factors, including pH, type, and concentration of carriers, affect the zeta potential value and surface charge [23]. 

### 3.2. Viability of Encapsulated L. acidophilus during Baking and Storage

The survival of *L. acidophilus* (log CFU/g) in bread before and after encapsulation in alginate/fish gelatin during the baking process and 7 days storage are presented in Table 2. The survival of *L. acidophilus* in all samples finally may be expected to decrease due to the high temperature during baking [24]. The results indicated that bread baking at 175 °C for 6 min reduced the viable cell count of *L. acidophilus* by 3.95–6.51 log CFU/g. However, the reduction in *L. acidophilus* during baking was much lower in the encapsulated cells in alginate/fish gelatin 1.5 and 3%, which might be attributed to the heat-protective effects of alginate and fish gelatin on the *L. acidophilus* cells. The survival of probiotics in bread during baking and storage is significantly influenced by the incorporated probiotic strains and physicochemical properties of capsule materials [4]. In a similar study, Zhang et al. [4] observed that the survival of *L. plantarum* significantly decreased to 104~5 CFU/g after bread baking at 180 °C for 5 min. Our results agree with Thang et al. [25], showing the viability of *L. acidophilus* encapsulated in a combination of alginate and xanthan gum was decreased by 3.64 log CFU/g during baking compared to non-capsulated, which was about reduced 5 logs CFU/g. 

Re-growth of *L. acidophilus* was observed during the 7-day storage of bread. The bread matrix is an appropriate environment for the growth of probiotic bacteria because of the abundance of nutrients for bacterial. However, the increase in probiotic bacteria counts during the first day was insignificant (*p* > 0.05). This lag phase indicates that surviving cells need time to adapt to the new condition and cellular damage recovery [26]. After this lag phase, the viable counts of *L. acidophilus* increased by 0.5–1.5 in 4-day storage. The viability of *L. acidophilus* encapsulated by alginate/fish gelatin 1.5 and 3% achieved the suggested concentration (10^6^ CFU/g) for probiotic food products. After 7-day storage, encapsulation of *L. acidophilus* by alginate as the single carrier and in combination with fish gelatin (0.5, 1.5, and 3%) increased the viability by about 1 and 1.5–3 log cycles, respectively. The study of Trabelsi et al. [20] indicated that the protective effect of alginate in combination with polymer compounds on the *L. plantarum* viability showed better results than using alginate alone during 35 days of storage at 4 °C.

### 3.3. Hardness and Staling Rate

**Figure 1:**
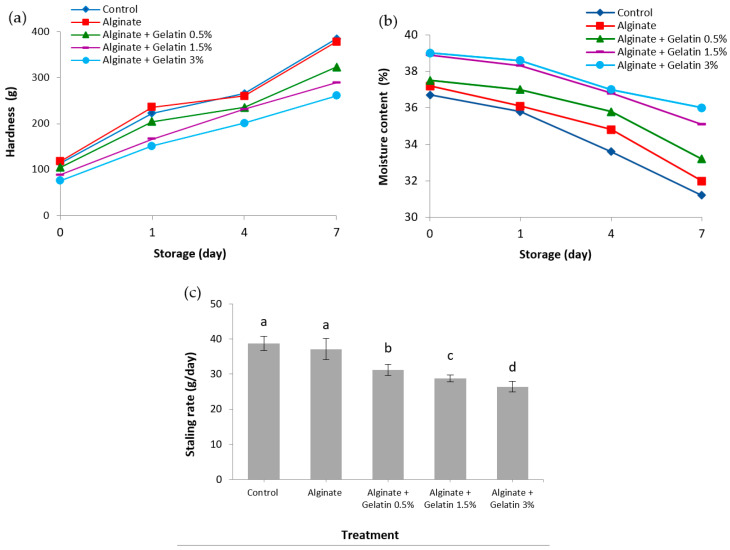
(**a**) The hardness, (**b**) moisture content, and (**c**) staling rate bread supplemented with encapsulated *L. acidophilus* in different matrices during storage. Means ± SD with different letters in the same column represent significantly different (*p* > 0.05).

**Figure 2:**
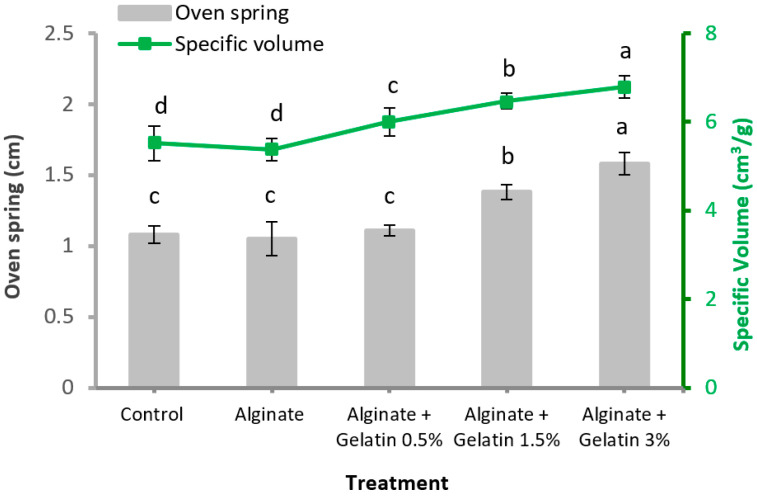
Oven spring and specific volume of bread supplemented with encapsulated *L. acidophilus* in different matrices. Means ± SD with different letters in the same column or line represent significantly different (*p* > 0.05).

The effects of free and encapsulated *L. acidophilus* in alginate individually and in combination with fish gelatin at 0.5, 1.5, and 3% on hardness and staling rate of bread during storage are shown in Figure 1a,c. The hardness of bread significantly increased during 7-day storage. However, fish gelatin decreased crumb hardness, in which the crumb hardness with encapsulated *L. acidophilus* in alginate/fish gelatin 3% was 32.2% less than that of control bread after 7-day storage. This result was probably related to more moisture content and a larger specific volume of bread caused by the increment of fish gelatin (Figure 1b and Figure 2). The staling rate of bread containing encapsulated *L. acidophilus* in alginate/fish gelatin 0.5, 1.5, and 3% decreased by 19.5, 25.8, and 31.7%, respectively. It appears that the weakening effect of hydrocolloids on the starch structure of bread leads to a decrease in crumb resistance and better water retention and distribution. A similar study has presented that some hydrocolloids could also reduce the hardness during bread storage [27]. By preferential binding hydrocolloids to starch, retrogradation of amylopectin is inhibited, thus hardening is postponed [28]. In a similar study, Yu et al. [11] observed that the hardness of both fresh and stored bread significantly decreased with the addition of pigskin gelatin (1%) in bread, achieving a decrease (20.8%) in staling rate for bread after 7 days of storage.

### 3.4. Moisture Content

Moisture loss, distribution of moisture between the crystalline and amorphous zone, and hardness must participate in the bread staling process [29]. The moisture content of bread supplemented with encapsulated *L. acidophilus* in different matrices during storage is shown in Figure 1b. The highest moisture content was found in the bread supplemented with encapsulated *L. acidophilus* in alginate/fish gelatin 3% matrix, followed by the alginate/fish gelatin 1.5% matrix. These finds were ascribed to the water-holding capacity of the fish gelatin. The jelly texture of fish gelatin allows it to keep the water in its matrix and have more water throughout the heating process. Gelatin demonstrates barrier capabilities to prevent the removal of water during the baking process by making water molecular hydrogen bonds, which make them more effective to avoid weight loss in the final product [30]. Lafarga et al. [31] similarly found that chitosan generated from marine shellfishery byproducts improved the moisture content for fresh bread and decreased moisture content after 5 days of storage.

### 3.5. Oven Spring and Specific Volume 

Figure 2 illustrates the oven spring and specific volume values of bread supplemented with encapsulated *L. acidophilus* in different matrices. The results indicated that the oven spring and specific volume values of bread ranged from 1.05 to 1.58 cm and 5.38 to 6.79 cm^3^/g, respectively. As shown in Figure 2, the addition of alginate-encapsulated *L. acidophilus* to bread had no significant effect on both the oven spring and specific volume index (*p* > 0.05). However, these values were significantly (*p* < 0.05) affected by the addition of alginate/fish gelatin-encapsulated *L. acidophilus*, indicating the positive effect of fish gelatin for improvement of oven spring and specific volume values of bread. The extensive interaction with fish gelatin due to hydroxyl groups bonding with hydrogen on water may allow the dough to tolerate the significant increases in water absorption, leading to increased volume. The rise in oven spring and specific volume values of bread could be due to increased instability of the gluten–starch network, dough strength, and gas retention capacity [4,32]. Yu et al. [11] reported the bread volume significantly enhanced (*p* < 0.05) with the increment of 1% gelatin pigskin, which depends on the gas holding capacity of dough. 

### 3.6. Internal Texture Structure

**Figure 3:**
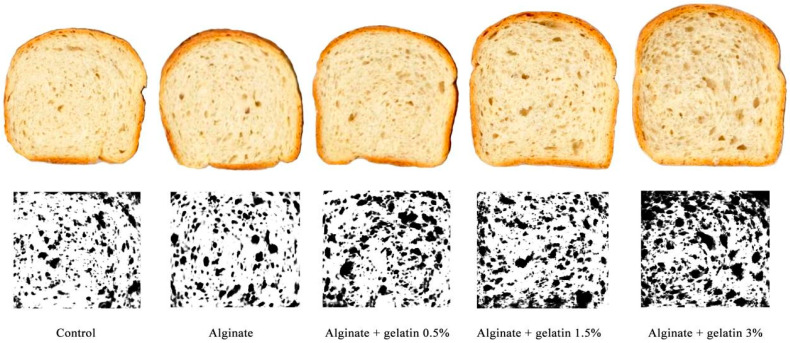
The morphological profiles of cell structure from bread supplemented with encapsulated *L. acidophilus* in different matrices.

Figure 3 crumb images of the bread samples and the binarized images are used to compute the morphological characterizations of the internal texture structures. Moreover, the image analysis results of crumb structures (porosity, cell average area, and cell density) of bread supplemented with encapsulated *L. acidophilus* in different matrices are shown in Table 3. Compared to the control sample, bread supplemented with alginate-encapsulated *L. acidophilus* had a similar porosity and a lower cell average. Fish gelatin had a favorable influence on the specific volume value of bread, which relies on the gas-holding capacity of dough. The enhanced gas-holding capacity resulted in higher porosity in the bread supplemented by fish gelatin, higher cell density, and a smaller cell area. The density and size of the gas cells could lead to enormous textural and sensory attributes of the bread [11]. As shown in Figure 3, the size and height of the bread are increased by the addition of fish gelatin, which agrees with the obtained results of the oven spring and specific volume in the previous section (Section 3.5). A dual mechanism can explain gas cell stabilization at the molecular level: the gluten–starch matrix and thin liquid lamellae stabilized by adsorbed surface-active ingredients (proteins and lipids) at the gas–liquid interface. The former is the initial stabilizing factor to expand gas cells against coalescence and disproportionation [33,34]. 

## 4. Conclusions

The bread baking significantly decreased the survival of *L. acidophilus* to about 4.5 logs CFU/g in bread. The survival of encapsulated *L. acidophilus* during baking and storage is entirely affected by the composition of the capsule’s matrix. Encapsulation of probiotic cells by alginate/fish gelatin can increase their viability by 2.49 and 3.07 log CFU/g during baking and 7 days of storage, respectively. The alginate/fish gelatin 3% matrix exhibited the highest protective effect on *L. acidophilus* cells during baking and storage of bread. Fish gelatin as a second layer carrier of the bacteria had a positive effect on improving the technical quality of bread. Overall, the findings suggest encapsulation of *L. acidophilus* in alginate/fish gelatin capsule has great potential to improve probiotic bacteria’s survival during baking and storage and serves as an effective bread enhancer.

## Figures and Tables

**Table 1:** Encapsulation efficiency, particle size, and zeta potential of capsules with different matrices.

Sample	Encapsulation Efficiency (%)	Particle Size (µm)	Zeta Potential (mV)
Alginate	92.30 ± 3.18 ^d^	389.8 ± 20.9 ^d^	−13.5 ± 2.2 ^c^
Alginate + Gelatin 0.5%	95.42 ± 1.36 ^c^	569.3 ± 36.9 ^c^	−17.3 ± 1.4 ^b^
Alginate + Gelatin 1.5%	96.33 ± 2.45 ^b^	682.1 ± 0.05 ^b^	−21.0 ± 2.5 ^a^
Alginate + Gelatin 3%	98.68 ± 1.97 ^a^	736.5 ± 0.04 ^a^	−21.6 ± 3.1 ^a^

Means ± SD in the same column with different superscripts are significantly different (*p* > 0.05).

**Table 2:** The survival of *L. acidophilus* in bread before and after encapsulation in alginate/fish gelatin during the baking process and 7 days of storage (log CFU/g).

Sample	Storage (Day)				
	Dough	After Baking	1	4	7
Control	9.40 ± 0.29 ^a,A^	2.89 ± 0.13 ^c,D^	2.74 ± 0.14 ^c,D^	3.45 ± 0.16 ^c,C^	4.59 ± 0.11 ^c,B^
Alginate	9.22 ± 0.24 ^b,A^	4.43 ± 0.14 ^b,C^	4.36 ± 0.26 ^b,C^	5.28 ± 0.25 ^b,B^	5.44 ± 0.22 ^b,B^
Alginate + Gelatin 0.5%	9.35 ± 0.31 ^a,A^	4.37 ± 0.19 ^b,D^	4.20 ± 0.28 ^b,D^	5.15 ± 0.26 ^b,C^	5.57 ± 0.15 ^b,B^
Alginate + Gelatin 1.5%	9.19 ± 0.12 ^b,A^	5.21 ± 0.25 ^a,D^	5.19 ± 0.15 ^a,D^	6.25 ± 0.12 ^a,C^	7.82 ± 0.31 ^a,B^
Alginate + Gelatin 3%	9.33 ± 0.14 ^a,A^	5.38 ± 0.34 ^a,D^	5.25 ± 0.24 ^a,D^	6.42 ± 0.16 ^a,C^	7.66 ± 0.24 ^a,B^

Means followed by the different lowercase letters in a column and capital letters on the lines are significantly different (*p* < 0.05).

**Table 3:** Morphological characterizations of crumb structures of bread supplemented with encapsulated *L. acidophilus* in different matrices.

Sample	Porosity (%)	Cell Average Area (mm^2^)	Cell Density (Cells/cm^2^)
Control	30.49 ± 3.11 ^d^	0.69 ± 0.06 ^d^	37.36± 4.24 ^c^
Alginate	31.25 ± 1.89 ^d^	0.60 ± 0.03 ^c^	41.96 ± 2.16 ^b^
Alginate + Gelatin 0.5%	34.73 ± 2.05 ^c^	0.55 ± 0.07 ^b,c^	43.01 ± 5.50 ^b^
Alginate + Gelatin 1.5%	38.19 ± 3.64 ^b^	0.51 ± 0.05 ^b^	47.83 ± 2.95 ^a^
Alginate + Gelatin 3%	41.25 ± 3.37 ^a^	0.43 ± 0.04 ^a^	48.20 ± 3.46 ^a^

Means ± SD with different letter in the same column represent significantly different (*p* > 0.05).

## Data Availability

The data presented in this study are available on request from the corresponding authors.
